# *Candida krusei* form mycelia along agar surfaces towards each other and other *Candida* species

**DOI:** 10.1186/s12866-017-0972-z

**Published:** 2017-03-11

**Authors:** Jacob Fleischmann, Corey D. Broeckling, Sarah Lyons

**Affiliations:** 1 David Geffen School of Medicine at UCLA, Los Angeles, CA USA; 2 Research Division of GLA VA, Los Angeles, CA USA; 3 Proteomics and Metabolomics Facility, Colorado State University, Fort Collins, CO USA

## Abstract

**Background:**

*Candida krusei* has been known to exhibit communal interactions such as pellicle formation and crawling out of nutritional broth. We noticed another possible interaction on agar surfaces, where *C. krusei* yeast cells formed mycelia along agar surfaces toward each other. We report here the results of experiments to study this interaction.

**Results:**

When *C.krusei* yeast cells are plated in parallel streaks, they form mycelia along agar surfaces toward other yeasts. They also detect the presence of *Candida albicans* and *Candida glabrata* across agar surfaces, while the latter two react neither to their own kind, nor to *C. krusei.* Secreted molecule(s) are likely involved as *C.krusei* does not react to heat killed *C. krusei.* Timing and rate of mycelia formation across distances suggests that mycelia start forming when a secreted molecule(s) on agar surface reaches a certain concentration. We detected farnesol, tyrosol and tryptophol molecules that may be involved with mycelial formation, on the agar surfaces between yeast streaks. Unexpectedly the amounts detected between streaks were significantly higher than would have expected from additive amounts of two streaks. All three *Candida* species secreted these molecules. When tested on agar surface however, none of these molecules individually or combined induced mycelia formation by *C. krusei*.

**Conclusions:**

Our data confirms another communal interaction by *C. krusei,* manifested by formation of mycelia by yeast cells toward their own kind and other yeasts on agar surfaces. We detected secretion of farnesol, tyrosol and tryptophol by *C. krusei* but none of these molecules induced this activity on agar surface making it unlikely that they are the ones utilized by this yeast for this activity.

## Background

*Candida* species have grown in importance as human pathogens, now being the fourth most common organisms isolated from blood cultures [18]. *Candida krusei* while representing a small percentage of these isolates, has become important as a pathogen for immunocompromised patients, especially because of its intrinsic resistance to some anti-fungal agents [17]. It also has been recognized to be playing a role in food fermentation [20] especially in the production of cocoa [11]. Interesting interactions among these yeasts have been described. For example, quorum sensing (QS), a synchronous expression of molecules as a function of population density, originally described in bacteria [13] has been found to be present in *Candida* species [1]. At high population densities *Candida albicans* produces farnesol which inhibits hyphal transformation, a major feature of this yeast [10]. Other alcohols with quorum sensing activities in *C. albicans* include tyrosol [5] and tryptophol [8]. Unusual communal behavior has also been described for *C. krusei*. It forms pellicles across the broth as it grows and also has the capacity as a yeast to crawl out of the nutritional broth along the inner side of tubes [19]. We have reported this phenomenon to be useful in the phenotypic identification of this organism [7]. Colonies growing on agar surface are known to eventually form mycelial elements at the periphery [16]. We now report interesting features to this mycelial formation on agar. It appears that it senses the presence of its own kind and other *Candida* species across the agar surface.

## Methods

### Organisms and chemicals

*Candida krusei* ATCC 14243, *Candida albicans* SC5314, ATCC MYA 2876, *Candida glabrata* ATCC MYA 2950 and were maintained in 50% glycerol in YPD broth (2% w/V peptone, 1% w/v yeast extract, 2% w/v dextrose) at −80 °C. Cells were grown in YPD broth at 30 °C and maintained on Sabouraud’s dextrose agar at 4 °C, passaged every 4–6 weeks up to 4–5 times. Additional clinical isolates of *C. krusei* identified as such by MALDI-TOF, were obtained from the UCLA Clinical Microbiology Laboratory. Farnesol (F203), tyrosol (188255) and tryptophol (T90301) were all obtained from Sigma.

### Agar surface assay

**Fig. 1:**
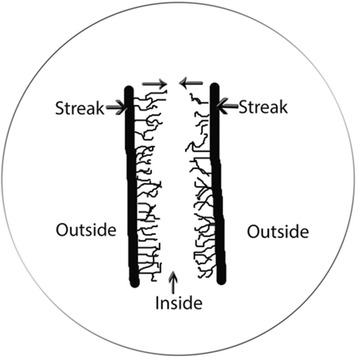
Drawing representing the basic elements of agar surface assay

Unless otherwise noted organisms were lifted from agar surface with 2 mm sterile loop and streaked unto Sabouraud’s dextrose agar in parallel (Fig. 1). Distances between streaks were as indicated in results. Any growth activity between parallel streaks is referred to as “inside” and those not in-between are referred to as “outside”. Non-viable controls were yeast grown in YPD broth at 30 °C, autoclaved and spun down and a loopful streaked from the pellet. Plates were incubated at 30 °C and observed daily for any mycelial formations, either with an inverted scope or a regular microscope at 4× and 10× magnification. Pictures were obtained with a Canon Vixia HF S30 camera adapted to microscope eyepiece tube with a MM99 adapter S/N:5343 (Martin Microscope Co). To look at possible effect of number of yeasts streaked, a single colony was grown overnight in YPD with shaking at 30 °C and a loopful from this mixture or from a 1:10 dilution into fresh YPD broth were streaked.

### Gas Chromatography Mass Spectrometry (GC-MS)

Organisms were streaked 10 mm apart and grown at 30 °C for three days. Gel pieces were punched out with sterile pipette tips that were shortened with sterile blades to give the same sized opening. They were obtained from either between parallel streaks (Fig. 1. – inside, about 5 mm from streaks) or outside of parallel streaks (Fig. 1. – outside, about 5 mm from streak) under inverted microscope visualization to avoid organisms. They were immediately frozen at −20 °C pending analysis for secreted molecules. Pieces from agar without streaked organisms were controls.

Gel pieces were placed into 2.0 mL tubes, and 1 mL of 80% methanol was added to the plug, containing 1 μg of 13C Glucose (internal standard). For the agar only sample, it was cut into pieces of similar size to the remaining samples. The sample was extracted in a sonicating water bath for 15 min, then vortexed at high speed and room temperature for 15 min. This process was repeated 4 times to ensure quantitative extraction. The sample was then centrifuged at 14,000 × g at 4 °C, and 950 μL of the supernatant was transferred to a new tube. The solvent was removed under nitrogen, the extracted small molecules were derivatized in a two step derivatization process. Methoximation was performed to stabilizing carbonyl functional groups using 20 mg/mL methoxyamine hydrochloride in pyridine, incubating at 37 °C for 45 min, with intermittent sonication and vortexing. Subsequently, 50 μL of MSTFA + 1% TMCS was added to trimethylsilylate amine, carboxylic acid, and hydroxyl functional groups of the metabolites. Standard 1 mg/mL stock solutions were prepared in methanol, and a dilution series was made from 0.1 to 0.0001 mg/mL. Each curve contained 13C glucose (internal standard).

### GC-MS data acquisition

Metabolites were detected using a Trace GC Ultra coupled to a Thermo ISQ mass spectrometer (Thermo Scientific). Samples were injected in a 1:10 split ratio twice in discrete randomized blocks. Separation occurred using a 30 m TG-5MS column (Thermo Scientific, 0.25 mm i.d., 0.25 μm film thickness) with a 1.2 mL/min helium gas flow rate, and the program consisted of 80 °C for 30 s, a ramp of 15 °C per min to 330 °C, and an 8 min hold. Masses between 50 and 650 m/z were scanned at 5 scans/sec after electron impact ionization.

### Data processing

All quantitative data were processed using Thermo XCalibur Quan. Selected ions were chosen for each target, and peak areas were extracted. These areas were calibrated to concentration using the dose-response calibration curve as described above.

### Agar assay testing with alcohols

Stock solutions of 10 mg/ml for farnesol, tyrosol and tryptophol were prepared in ethanol. Stock solutions were serially diluted into fresh YPD broth at 1:10 ratios, and 50 μL of each concentration was spread on part Sabouraud’s dextrose agar surface and allowed to get absorbed. *C. krusei* were streaked starting from alcohol free surface (no alcohol control) continued over the alcohol containing surface. For some experiments the alcohols were diluted into melted SBA to a final concentration of 1 mg/ml and allowed to solidify and were streaked with organisms. For controls, single and parallel streaks were placed on alcohol-free SBA. Plates were incubated at 37 °C and observed daily for mycelia formations.

## Results

### Agar surface assays with *C. krusei* vs *C. krusei*

**Fig. 2:**
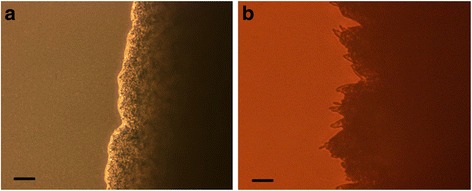
Single streak of *C. krusei* on Sabouraud’s dextrose agar incubated at 30 °C, **a** - after 3 days, **b** – after 5 days. For both, only one side is shown. Even at 5 days, early mycelia can be seen only in relatively small numbers. Scale bars (SB) = 100 μm

**Fig. 3:**
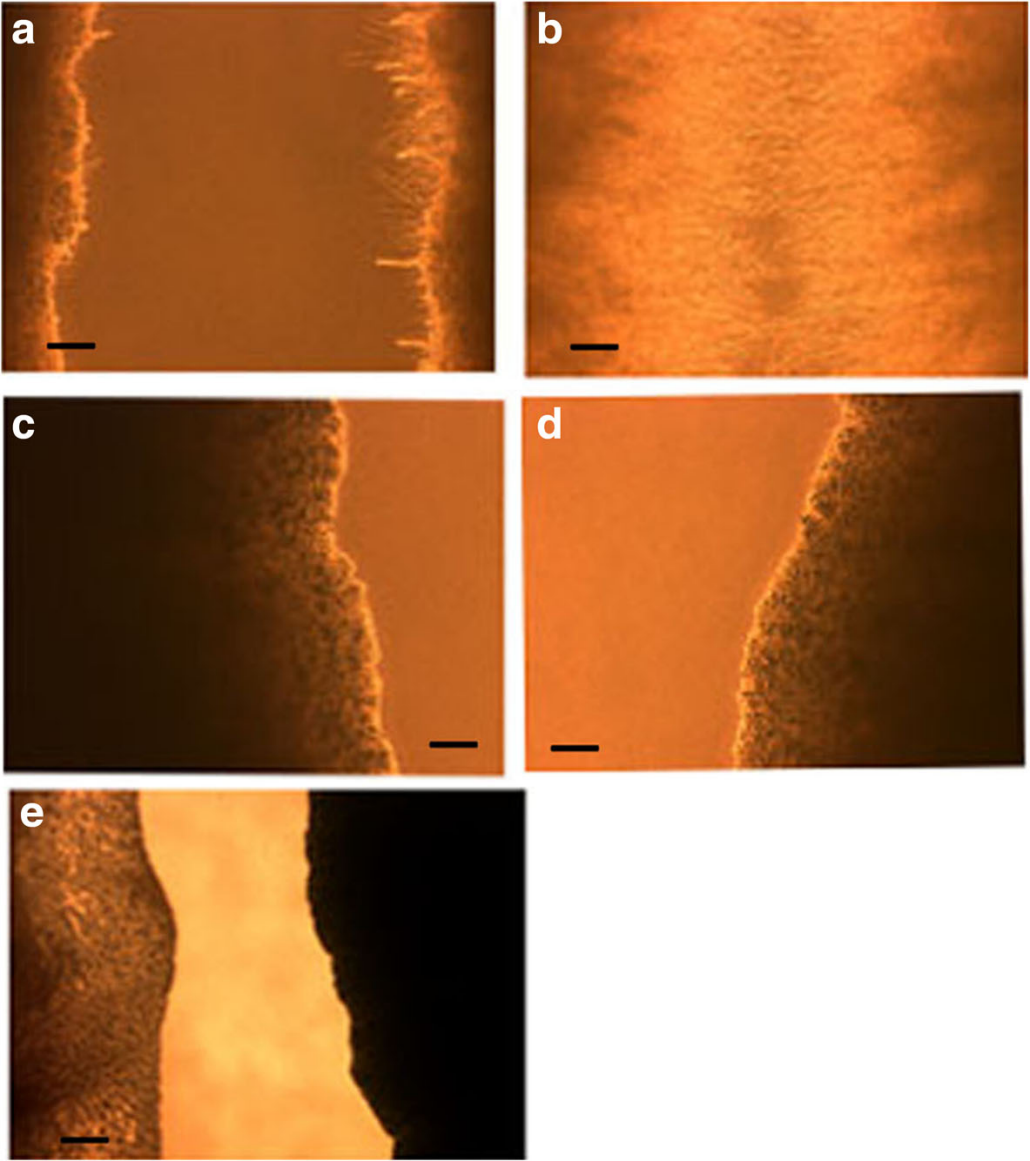
Parallel streaks of *C. krusei* 5 mm apart on Sabouraud’s dextrose agar incubated at 30 °C. **a** – after 2 days some early mycelia. **b** – after 3 days heavy inside mycelial rowth. **c** and **d** - outer sides of both streaks after 3 days show no outside mycelial growth. Inside and outside refer to setup shown on Fig. 1. **e** – Parallel streaks of *C. krusei*, viable (*left side*) vs. heat killed (*right side*) after 3 days, no mycelia are formed. SB = 100 μm

As mentioned previously, *C. krusei* has the capacity to crawl out of incubation solution [19]. Geotropism, the capacity to respond either toward or away from gravitational force has been described for fungi [14], thus we wondered if this capacity to crawl out of solution had such a basis. To see if mycelial formation will respond similarly, we streaked out yeast cells parallel to each other on Sabouraud’s dextrose agar (SDA) as illustrated on Fig. 1 and maintained the Petri dish sideways at 30 °C with the streaks being horizontal one over the other. What we saw was unexpected, as after several days we observed mycelia forming along sides of streaks facing each other and not the outer sides (Fig. 1). This suggested that the organisms were “sensing” each other across the agar surface between the streaks. All subsequent assays were done on agar plates incubated in the usual horizontal fashion. A single streak of *C. krusei* will take 5 or more days to form any mycelia at 30 °C on SDA surface (Fig. 2). When streaked in parallel, it consistently forms mycelia earlier than that on the sides facing each other, while the outer sides will take as long as single streaks. An example of this can be seen in Fig. 3, as after 2 days (a) mycelia can be seen forming on the facing inner surfaces and by the third day (b) the space is fully overgrown by mycelia. After three days, the outer surfaces (c, d) still show no evidence of mycelial growth. We have repeated this assay over 30 times with *Candida krusei* ATCC 14243 and it consistently behaved the same way each time. In addition, five separate clinical isolates of *C. krusei* behaved the same way.

That indeed they are reacting to other viable yeast cell can be seen in Fig. 3e. When viable yeast were streaked against non-viable ones (lighter viable, darker non-viable) no mycelia are seen after three days. The distance between streaks was 5 mm, but we saw this also in many assays where the distance was 10 mm. Distance between streaks did indeed make a difference as to the time it took for mycelia to appear and this is summarized on Table 1. In one experiment (data not shown), comparing one loop streaks from solution with yeast grown overnight and a 1:10 dilution of this solution, we found a more robust mycelia formation from the higher number of yeast streaked, as it related to timing and distance between streaks.

**Table 1:** Effects of distance and time on mycelia formation by *C. krusei*

Distance between streaks	Mycelia growth after		
	24 h	48 h	72 h
0.5 cm	0	++	++
1.0 cm	0	+	++
1.5 cm	0	0	+

0 = no mycelia

+ = mycelia present over less than half of streak

++ = mycelia present over more than half of streak

*n* = 2

### Agar surface assays with *C. krusei* vs. other *Candida* species

**Fig. 4:**
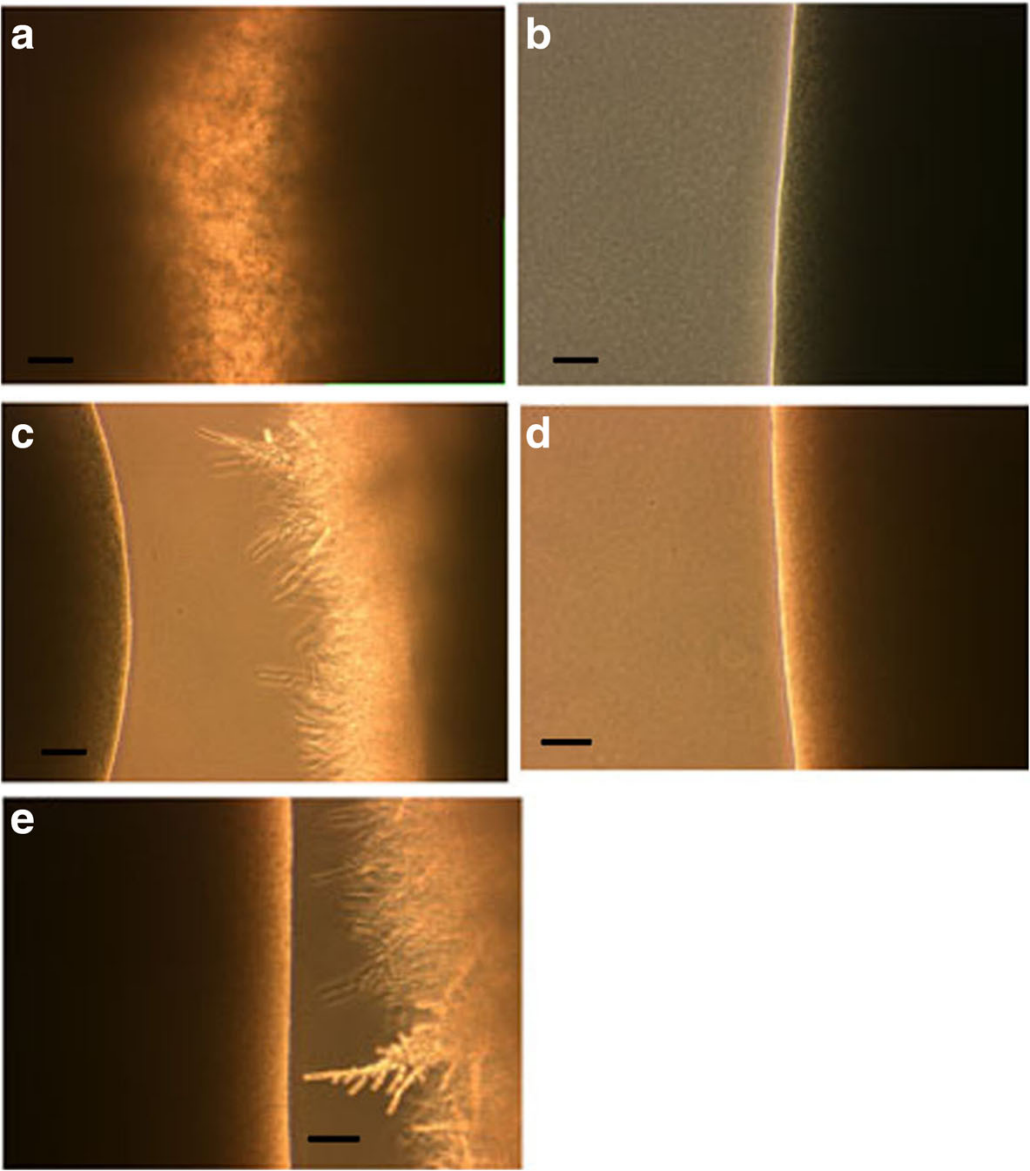
Parallel streaks 5 mm apart, of *C. krusei* vs. *C. albicans* or *C. glabrata.* **a** – *C. krusei* vs. *C. krusei* after 3 days with robust mycelial growth as control. **b** – *C. albicans* vs. *C. albicans* after 3 days no mycelia are formed (only one of the inner sides shown). **c** – *C. albicans* (left) vs. *C. krusei* (right) showing no mycelial growth by *C. albicans* and robust mycelial growth by *C. krusei* after 3 days. **d** – *C.glabrata* vs. *C. glabrata* after 3 days no mycelia are formed (only one of the inner sides shown). **e** – *C. glabrata* (*left*) vs. *C. krusei* (*right*) showing no mycelial growth by *C. glabrata* and robust mycelial growth by *C. krusei* after 3 days. SB = 100 μm

To see if this capacity to detect other yeast is specific for *C. krusei* or it can also sense other *Candida* species, we chose *C. albicans* a robust germinator and *C. glabrata* not known to germinate under usual growth conditions though a pseudohyphae-like growth when starved [6] or when exposed to macrophages have been described for it [4]. Figure 4 represents the results with *C. krusei* vs. *C. albicans* and *C. glabrata.* At a distance of 5 mm between streaks by the third day (a) *C. krusei* vs. *C. krusei* show robust mycelia between them. In contrast, *C. albicans* vs. *C. albicans* for the same length of time (b) both inner sides show no mycelia (only one shown). In sharp contrast (c) *C. krusei* vs. *C. albicans* by day three, *C.* krusei is showing significant mycelia while *C. albicans* (left side of c) shows no mycelia. Similar results are seen with *C. krusei* vs. *C. glabrata.* On *C. glabrata* vs. *C. glabrata* (d – only one side shown) no mycelia are seen, while *C. krusei* vs. *C. glabrata* (e) *C. krusei* is germinating while *C. glabrata* (left side of e) is not. These results are summarized on Table 2.

**Table 2:** Summary of mycelia formation between streaks of pairs of *Candida* species

Organisms^a^	72 h
*C. krusei*	++
*C. krusei*	++
*C. albicans*	0
*C. albicans*	0
*C. glabrata*	0
*C. glabrata*	0
*C. krusei*	++
*C. albicans*	0
*C. krusei*	++
*C. glabrata*	0

++ = more than 50% of the streak forming mycelia for the specific yeast

0 = no mycelia formed by yeast

^a^Pairs represent candida species streaked parallel to each other

### Analysis for secreted molecules on agar surface

**Fig. 5:**
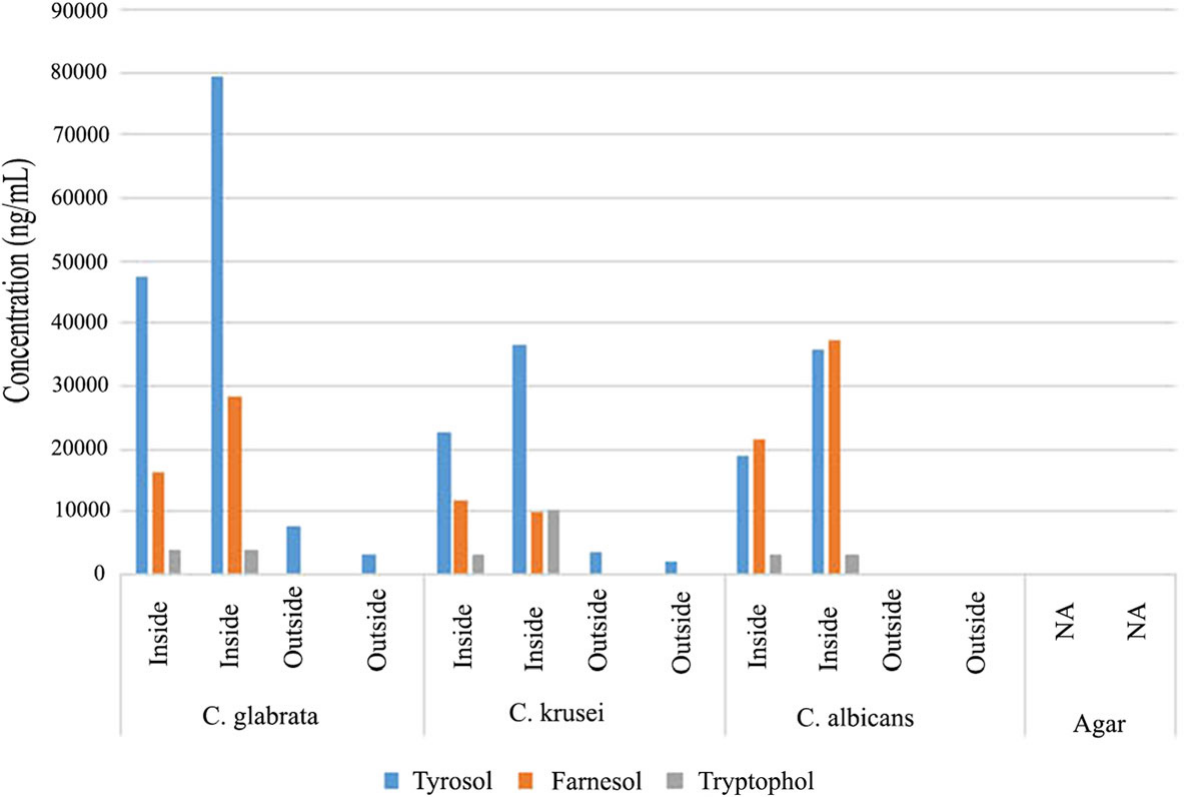
Quantities of tyrosol, farnesol and tryptophol produced by *Candida* species on agar surface as measured by GM-CS. Distance between streaks was 10 mm and inside and outside refer to setup shown on Fig. 1 (see text for more details). Agar without any yeast growing on it resulted in none of the 3 alcohols detected

Without focusing on specific target molecules, GC-MS analysis detected a very high number of molecules on agar surface by *C. krusei*, making it impractical to identify the one or ones involved in this sensing activity. As our surface assays suggested that *C. krusei* reacted to something produced by *C. albicans*, we chose three molecules well established to be secreted by *C. albicans.* The three we looked at are farnesol, tyrosol and tryptophol. The quantitative results for all three are depicted in Fig. 5. All three yeasts produced all three alcohols with some variations. Tyrosol was the most produced by *C. krusei* and *C. glabrata* significantly more than farnesol, while *C. albicans* produced them in nearly equal amounts. The least produced by all three was tryptophol. Consistent for all three alcohols was the finding that much more could be detected between the streaks (10 mm apart) than outside of them, by day three. No detection on agar alone clearly confirms yeast secretions.

### Agar assay with alcohols

To see these if these described quorum sensing alcohols played a role in mycelial formation, they were tested individually, in combinations of two and three and at various concentrations. At no time did we observe any mycelia formation on a single streak any sooner than the single control. The parallel streaks on agar alone formed mycelia as usual.

## Discussion

Our observation of *C. krusei* sensing each other on agar surface, points to another interesting phenotypic behavior by this yeast in addition to pellicle formation on broth surface and crawling out of broth. The fact that differences in distances between streaks affected the time of appearance in mycelia is consistent with a molecule (or molecules) secreted by the yeast along the agar surface that induces mycelial growth when it reaches a certain concentration. Our data also shows that this is not limited to self-recognition as it also reacts to *C. albicans* and *C. glabrata*. At least among these three yeasts, it seems to be unique to *C. krusei* as neither of the other two react to themselves nor to *C. krusei*. Mycelial formation by *C. krusei* appears to be specific for agar surface as we have not observed any such activity either in YPD broth or in serum, the latter being a potent inducer of hyphal formation for *C. albicans* [3]. Thigmotropism, growth along surfaces in response to contact has been described for *C. albicans* [12] and may play a role in this behavior by *C. krusei* but clearly our data shows it to be focusing at specific targets on the agar surface, which requires more than contact stimuli. Not unexpectedly, unfocused GC-MS disclosed many molecules, some of which surely originated from the agar. If one of the three molecules we detected did mediate this process, it is interesting that all three yeasts are producing it, yet only one of them, namely *C. krusei* is reacting to it. Other possible less specific triggers include pH changes at agar surface and depletion of nutrients causing *C. krusei* to form mycelia to seek nutrients.

Among the alcohols we looked at, farnesol actually inhibited yeast-to-mycelium transition in *C. albicans* [10]. Once the cells were already committed to mycelial formation however, it no longer inhibited them [15]. On the other hand tyrosol stimulates filamentation [2]. The role for tryptophol in filamentation remains unclear [1]. Our GC-MS data clearly establishes the secretion of these alcohols on agar surface by all three yeasts, but we could not demonstrate any role for them inducing filamentation across agar surface. Another yeast, important in food production *Debaryomyces hansenii* similarly produced tyrosol with an effect on some functions such as adhesion and slide motility but had no effect on pseudohyphal growth [9].

The one unexpected and interesting finding regarding the alcohol production by these yeasts is the quantitative difference between agar surfaces between streaks versus those outside (Fig. 5). Assuming no additional stimuli, a streak of cells would be expected to produce these molecules and secrete them along both sides onto agar surfaces at the same rate. The samples obtained were five millimeters from the streak outside and halfway between streaks which is also five millimeters from each streak. Thus if these streaks are indeed secreting these alcohols at an equal rate we would expect the amount measured inside to be additive and to be about double the amount measured on the outside. What we see is quite different. For example, for *C. albicans*, neither farnesol nor tyrosol could be detected outside while in between streaks significant amounts were produced. For *C. krusei* and *C. glabrata* the predominant molecule produced is tyrosol. While some could be detected outside, the ratio of inside production to outside is well above 2:1. It appears that alcohol production between streaks is boosted in a synergistic fashion. This appears to mirror mycelial formation (Fig. 2) as by the second day mycelial formation are clearly visible on the inner side of the streaks (a) and robustly by the third day (b). Yet on the outer sides of the streaks, no mycelial formations are present after the third day and usually we would see some starting to develop only after five days. One possibility is that mycelia may be the more efficient producers of these alcohols and therefore their production is a result of the inner robust mycelia formation and not happening parallel to it.

## Conclusions

That *C. krusei* reacts to itself and *C. albicans* and *C. glabrata* across agar surfaces, while these two do not react to themselves or *C. krusei* is an unexpected finding. Since this phenomenon is manifested by mycelial formation, identifying the underlying mechanism for it should add significantly to our understanding of hyphal transformation. While we found production of farnesol, tyrosol and tryptophol by all three yeasts on agar surface, these molecules did not mediate this response. However their production exhibits an interesting quantitative pattern, accumulating to more than additive higher concentrations between parallel streaks than outside them.
